# Supplementary material for: The Significance of Systemic Inflammation Markers in Intrahepatic Recurrence of Early-Stage Hepatocellular Carcinoma after Curative Treatment
Source: Cancers (Basel). 2022 Apr 21;14(9):2081. doi: 10.3390/cancers14092081 (PMC9102776; doi:10.3390/cancers14092081)
Supplement: Supplementary file 1 [file cancers-14-02081-s001.zip › cancers-1668036-supplementary.pdf]

cancers

MDPI

Supplementary Material: The Significance of Systemic Inflammation Markers in Intrahepatic Recurrence of Early-Stage Hepatocellular Carcinoma after Curative Treatment

Bong Kyung Bae, Hee Chul Park *, Gyu Sang Yoo, Moon Seok Choi, Joo Hyun Oh and Jeong Il Yu *

Supplemental Material

Table S1. Univariable and multivariable cox regression analysis of intrahepatic recurrence free survival for patients who received hepatic resection or RFA as initial treatment.

Factors	Univariable		Multivariable	
	HR (95 % CI)	*p*-value	HR (95 % CI)	*p*-value
Sex (Male)	1.207 (1.082–1.345)	0.001	1.240 (1.108–1.389)	<0.001
Age (>60)	1.071 (0.978–1.173)	0.138	1.134 (1.033-1.246)	0.008
Viral status (Viral)	1.158 (1.016–1.321)	0.028		
AST (>40)	1.590 (1.457–1.735)	<0.001		
ALT (>40)	1.294 (1.184–1.415)	<0.001		
ALBI grade (2–3)	1.659 (1.515–1.818)	<0.001	1.211 (1.095–1.340)	<0.001
Child-Pugh score (6–9)	1.435 (1.292–1.594)	<0.001		
AFP (>100)	1.014 (0.917–1.123)	0.783		
n of tumor (2–3)	1.704 (1.487–1.952)	<0.001	1.324 (1.146–1.529)	<0.001
Tumor size (>5cm)	1.058 (0.898–1.246)	0.502		
BCLC stage (A)	1.141 (1.043–1.249)	0.004	1.158 (1.050–1.277)	0.003
Platelet (≤150,000)	1.583 (1.445–1.733)	<0.001	1.123 (1.002–1.259)	0.046
ANC (≤ 2,500)	1.334 (1.223–1.455)	<0.001		
ALC (>1,200)	1.190 (1.067–1.327)	0.002	1.147 (1.014–1.298)	0.029
AMC (≤500)	1.071 (0.970–1.182)	0.176	1.158 (1.037-1.292)	0.009
NLR (>1.0)	1.037 (0.936–1.149)	0.487		
PLR (≤ 65.0)	1.152 (1.056–1.257)	0.002	1.162 (1.050–1.285)	0.004
LMR (≤4.0)	1.173 (0.948–1.312)	0.155		
ALRI (>15.0)	1.666 (1.504-1.845)	<0.001	1.503 (1.339-1.688)	<0.001
Change in NLR (>1.1 fold)	2.395 (2.194–2.614)	<0.001	1.515 (1.358–1.691)	<0.001

Change in PLR (>1 fold)	2.651 (2.428–2.895)	<0.001	1.964 (1.765–2.184)	<0.001
Change in LMR (≤1 fold)	2.237 (2.048–2.443)	<0.001	1.605 (1.452–1.774)	<0.001
Change in ALRI (>1.1 fold)	1.191 (1.092-1.299)	<0.001		

RFA, radiofrequency ablation; AST, aspartate transaminase; ALT, alanine transferase; ALBI, albumin-bilirubin; AFP, alpha-fetoprotein; *n*, number; BCLC, Barcelona Clinic Liver Cancer; PLT, platelet; ANC, absolute neutrophil count; ALC, absolute lymphocyte count; AMC, absolute monocyte count; NLR, neutrophil-to-lymphocyte ratio; PLR, platelet-to-lymphocyte ratio; LMR, lymphocyte-to-monocyte ratio; ALRI, AST-to-lymphocyte ratio index.

Table S2. Univariable and multivariable results of binary logistic regression analysis for potential factors of early and late intrahepatic recurrence for patients who received hepatic resection or RFA as initial treatment.

Factors	Early IHR				Late IHR			
	Univariable		Multivariable		Univariable		Multivariable	
	OR (95 % CI)	*p*-value	OR (95 % CI)	*p*-value	OR (95 % CI)	*p*-value	OR (95 % CI)	*p*-value
Sex (Male)	1.357 (1.120–1.643)	0.002	1.501 (1.213–1.857)	<0.001	1.292 (1.076–1.551)	0.006	1.499 (1.195–1.881)	<0.001
Age (>60)	0.894 (0.760–1.051)	0.174			0.883 (0.755–1.033)	0.883		
Viral status (Viral)	1.435 (1.144–1.799)	0.002			1.434 (1.153–1.783)	0.001		
AST (>40)	2.190 (1.863–2.575)	<0.001			1.757 (1.500–2.057)	<0.001		
ALT (>40)	1.693 (1.437–1.994)	<0.001			1.499 (1.278–1.759)	<0.001		
ALBI grade (2–3)	2.477 (2.079–2.950)	<0.001	1.612 (1.313–1.979)	<0.001	2.075 (1.747–2.465)	<0.001	1.539 (1.233–1.920)	<0.001
Child-Pugh score (6–9)	1.765 (1.447–2.154)	<0.001			1.580 (1.298–1.922)	<0.001		
AFP (>100)	1.192 (0.998–1.423)	0.052			1.185 (0.988–1.420)	0.067		
n of tumor (2–3)	2.461 (1.884–3.215)	<0.001	1.713 (1.258–2.333)	0.001	1.677 (1.267–2.218)	<0.001		
Tumor size (>5cm)	0.994 (0.758–1.303)	0.965			0.867 (0.738–1.125)	0.651		
BCLC stage (A)	1.294 (1.097–1.526)	0.002	1.231 (1.019–1.488)	0.031	0.895 (0.767–1.045)	0.160		
Platelet (≤150,000)	1.983 (1.688–2.328)	<0.001	1.278 (1.039–1.572)	0.020	1.986 (1.701–2.320)	<0.001	1.296 (1.037–1.619)	0.023
ANC (≤2,500)	1.620 (1.383–1.897)	<0.001			1.469 (1.260–1.711)	<0.001		
ALC (>1,200)	1.214 (0.993–1.483)	0.058			0.829 (0.682–1.006)	0.057	1.511 (1.158–1.972)	0.002
AMC (>500)	0.979 (0.818–1.173)	0.821			1.086 (0.915–1.290)	0.343		
NLR (>1.0)	1.025 (0.848–1.238)	0.799			0.858 (0.718–1.024)	0.090		
PLR (≤65.0)	1.206 (1.028–1.414)	<0.001	1.265 (1.035–1.547)	0.022	1.448 (1.243–1.688)	<0.001	1.421 (1.150–1.757)	0.001
LMR (≤4.0)	1.071 (0.876–1.310)	0.504			0.993 (0.816–1.209)	0.945		
ALRI (<15.0)	2.194 (1.857–2.592)	<0.001	2.266 (1.828–2.809)	<0.001	1.780 (1.488-2.131)	<0.001	1.860 (1.483-2.333)	<0.001
Change in NLR (>1.1 fold)	4.171 (3.516–4.947)	<0.001	2.274 (1.860–2.781)	<0.001	3.349 (2.837–3.954)	<0.001	2.041 (1.626–2.563)	<0.001

Change in PLR (>1 fold)	4.820 (4.069–5.710)	<0.001	3.128 (2.572–3.806)	<0.001	3.978 (3.378–4.684)	<0.001	2.928 (2.367–3.621)	<0.001
Change in LMR (≤ 1 fold)	3.563 (3.025–4.197)	<0.001	2.354 (1.946–2.848)	<0.001	2.770 (2.368–3.241)	<0.001	2.290 (1.863–2.814)	<0.001
Change in ALRI (>1.1 fold)	1.285 (1.109–1.489)	0.001			1.124 (0.954–1.324)	0.161		

RFA, radiofrequency ablation; AST, aspartate transaminase; ALT, alanine transferase; ALBI, albumin-bilirubin; AFP, alpha-fetoprotein; *n*, number; BCLC, Barcelona Clinic Liver Cancer; ANC, absolute neutrophil count; ALC, absolute lymphocyte count; AMC, absolute monocyte count; NLR, neutrophil-to-lymphocyte ratio; PLR, platelet-to-lymphocyte ratio; LMR, lymphocyte-to-monocyte ratio; ALRI, AST-to-lymphocyte ratio index

Table S3. Univariable and multivariable cox regression analysis of intrahepatic recurrence free survival for patients who received hepatic resection as initial treatment.

**Factors**	**Univariable**		**Multivariable**	
	**HR (95 % CI)**	*p*-value	HR (95 % CI)	***p*-value**
Sex (Male)	1.267 (1.080–1.485)	0.004	1.232 (1.042–1.456)	0.015
Age (>60)	0.979 (0.857–1.118)	0.752		
Viral status (Viral)	1.247 (1.035–1.503)	0.020		
AST (>40)	1.559 (1.376–1.767)	<0.001		
ALT (>40)	1.318 (1.163–1.494)	<0.001		
ALBI grade (2–3)	1.378 (1.193–1.590)	<0.001		
Child-Pugh score (6–9)	1.103 (0.924–1.316)	0.279		
AFP (>100)	1.044 (0.912–1.194)	0.533		
n of tumor (2–3)	1.379 (1.077–1.767)	0.011		
Tumor size (>5cm)	1.252 (1.054–1.489)	0.011	1.292 (1.072–1.557)	0.007
BCLC stage (A)	1.327 (1.140–1.543)	<0.001	1.340 (1.144–1.570)	<0.001
Platelet (≤150,000)	1.309 (1.158–1.481)	<0.001		
ANC (≤2,500)	0.921 (0.810–1.048)	0.213		
ALC (> 1,200)	0.922 (0.768–1.106)	0.380		
AMC (≤500)	1.094 (0.953–1.255)	0.202		
NLR (>1.0)	1.056 (0.917–1.216)	0.450		
PLR (≤65.0)	1.048 (0.922–1.191)	0.472		
LMR (≤4.0)	1.318 (1.152–1.508)	<0.001		
ALRI (>15.0)	1.514 (1.323–1.732)	<0.001	1.506 (1.303–1.740)	<0.001
Change in NLR (>1.1 fold)	2.633 (2.323–2.985)	<0.001	1.678 (1.431–1.967)	<0.001
Change in PLR (>1 fold)	2.950 (2.607–3.339)	<0.001	2.194 (1.892–2.545)	<0.001

Change in LMR (≤1 fold)	2.542 (2.243–2.881)	<0.001	1.947 (1.683–2.252)	<0.001
Change in ALRI (>1.1 fold)	1.182 (1.045–1.337)	0.008		

AST, aspartate transaminase; ALT, alanine transferase; ALBI, albumin-bilirubin; AFP, alpha-feto-protein; *n*, number; BCLC, Barcelona Clinic Liver Cancer; PLT, platelet; ANC, absolute neutrophil count; ALC, absolute lymphocyte count; AMC, absolute monocyte count; NLR, neutrophil-to-lymphocyte ratio; PLR, platelet-to-lymphocyte ratio; LMR, lymphocyte-to-monocyte ratio; ALRI, AST-to-lymphocyte ratio index.

Table S4. Univariable and multivariable results of binary logistic regression analysis for potential factors of early and late intrahepatic recurrence for patients who received hepatic resection as initial treatment.

Factors	Early IHR				Late IHR			
	Univariable		Multivariable		Univariable		Multivariable	
	OR (95 % CI)	*p*-value	OR (95 % CI)	*p*-value	OR (95 % CI)	*p*-value	OR (95 % CI)	*p*-value
Sex (Male)	1.287 (1.002–1.652)	0.048			1.324 (1.005–1.746)	0.046	1.492 (1.087–2.048)	0.013
Age (>60)	0.784 (0.635–0.968)	0.024			0.802 (0.637–1.009)	0.060		
Viral status (Viral)	1.684 (1.252–2.265)	0.001	1.796 (1.254–2.573)	0.001	1.363 (1.003–1.853)	0.048		
AST (>40)	2.239 (1.819–2.755)	<0.001	1.573 (1.176–2.104	0.002	1.622 (1.289–2.041)	<0.001		
ALT (>40)	1.746 (1.422–2.144)	<0.001			1.515 (1.209–1.898)	<0.001		
ALBI grade (2–3)	1.883 (1.467–2.418)	<0.001			2.096 (1.608–2.733)	<0.001	2.162 (1.470–3.181)	<0.001
Child-Pugh score (6–9)	1.404 (1.054–1.870)	0.020			0.902 (0.649–1.255)	0.541	1.905 (1.189–3.051)	0.007
AFP (>100)	1.146 (0.925–1.419)	0.213			0.881 (0.691–1.124)	0.308		
n of tumor (2–3)	1.488 (0.958–2.311)	0.077			1.763 (1.119–2.777)	0.014	1.678 (1.009–2.789)	0.046
Tumor size (>5cm)	1.374 (1.046–1.805)	0.022	1.435 (1.028–2.003)	0.034	0.882 (0.637–1.222)	0.450		
BCLC stage (A)	1.696 (1.312–2.193)	<0.001	1.487 (1.100–2.010)	0.010	0.906 (0.708–1.159)	0.432		
Platelet (≤150,000)	1.445 (1.181–1.767)	<0.001			1.768 (1.421–2.201)	<0.001		
ANC (≤2,500)	0.829 (0.672–1.023)	0.081			0.838 (0.666–1.053)	0.130		
ALC (>1,200)	0.854 (0.638–1.142)	0.287			1.030 (0.738–1.437)	0.863		
AMC (>500)	0.987 (0.788–1.236)	0.910			1.154 (0.908–1.466)	0.241		
NLR (>1.0)	1.062 (0.841–1.342)	0.612			0.810 (0.635–1.033)	0.089		
PLR (≤65.0)	1.141 (0.924–1.409)	0.221			1.272 (1.019–1.589)	0.034		
LMR (≤4.0)	1.409 (1.138–1.745)	0.002			1.161 (0.903–1.491)	0.244		
ALRI (<15.0)	1.903 (1.536–2.356)	<0.001	1.626 (1.194–2.214)	0.002	1.662 (1.321–2.091)	<0.001	1.920 (1.444–2.554)	<0.001
Change in NLR (>1.1 fold)	4.422 (3.528–5.527)	<0.001	2.402 (1.790–3.223)	<0.001	2.903 (2.273–3.709)	<0.001	1.604 (1.178–2.184	0.003
Change in PLR (>1 fold)	4.961 (3.980–6.184)	<0.001	3.315 (2.542–4.322)	<0.001	4.133 (3.260–4.241)	<0.001	3.454 (2.592–4.603)	<0.001

Change in LMR (≤1 fold)	3.696 (3.002–4.551)	<0.001	2.544 (1.979–3.270)	<0.001	2.799 (2.239–3.499)	<0.001	2.635 (2.000–3.472)	<0.001
Change in ALRI (>1.1 fold)	1.225 (1.004–1.495)	0.045			1.062 (0.854–1.321)	0.590		

AST, aspartate transaminase; ALT, alanine transferase; ALBI, albumin-bilirubin; AFP, alpha-fetoprotein; N, number; BCLC, Barcelona Clinic Liver Cancer; ANC, absolute neutrophil count; ALC, absolute lymphocyte count; AMC, absolute monocyte count; NLR, neutrophil-to-lymphocyte ratio; PLR, platelet-to-lymphocyte ratio; LMR, lymphocyte-to-monocyte ratio; ALRI, AST-to-lymphocyte ratio index.

Table S5. Univariable and multivariable cox regression analysis of intrahepatic recurrence free survival for patients who received RFA as initial treatment.

**Factors**	**Univariable**		**Multivariable**	
	**HR (95 % CI)**	***p*-value**	**HR (95 % CI)**	***p*-value**
Sex (Male)	1.215 (1.047–1.410)	0.010	1.288 (1.102–1.504)	0.001
Age (>60)	1.091 (0.962–1.237)	0.173	1.178 (1.036–1.340)	0.013
Viral status (Viral)	0.987 (0.821–1.186)	0.886		
AST (>40)	1.494 (1.321–1.690)	<0.001		
ALT (>40)	1.323 (1.165–1.502)	<0.001		
ALBI grade (2–3)	1.618 (1.430–1.831)	<0.001	1.289 (1.124–1.478)	<0.001
Child-Pugh score (6–9)	1.475 (1.290–1.688)	<0.001		
AFP (>100)	1.236 (1.057–1.445)	0.008	1.198 (1.021–1.406)	0.027
n of tumor (2–3)	1.613 (1.367–1.904)	<0.001	1.299 (1.076–1.569)	0.007
Tumor size (>5cm)	1.540 (0.385–6.169)	0.542		
BCLC stage (A)	1.628 (1.439–1.841)	<0.001	1.225 (1.060–1.415)	0.006
Platelet (≤150,000)	1.422 (1.213–1.668)	<0.001		
ANC (≤2,500)	1.318 (1.163–1.495)	<0.001		
ALC (>1,200)	0.968 (0.842–1.112)	0.646		
AMC (≤500)	1.112 (0.964–1.284)	0.145		
NLR (>1.0)	0.905 (0.779–1.052)	0.195		
PLR (≤65.0)	1.227 (1.085–1.388)	0.001		
LMR (≤4.0)	0.906 (0.799–1.027)	0.124	1.192 (1.038–1.369)	0.013
ALRI (>15.0)	1.606 (1.368–1.885)	<0.001	1.503 (1.263–1.787)	<0.001
Change in NLR (>1.1 fold)	1.845 (1.630–2.089)	<0.001	1.295 (1.123–1.494)	<0.001
Change in PLR (>1 fold)	2.019 (1.778–2.291)	<0.001	1.651 (1.428–1.909)	<0.001
Change in LMR (≤1 fold)	1.855 (1.639–2.101)	<0.001	1.427 (1.238–1.645)	<0.001

Change in ALRI (>1.1 fold)	1.200 (1.061–1.357)	0.004

RFA, radiofrequency ablation; AST, aspartate transaminase; ALT, alanine transferase; ALBI, albumin-bilirubin; AFP, alpha-fetoprotein; N, number; BCLC, Barcelona Clinic Liver Cancer; PLT, platelet; ANC, absolute neutrophil count; ALC, absolute lymphocyte count; AMC, absolute monocyte count; NLR, neutrophil-to-lymphocyte ratio; PLR, platelet-to-lymphocyte ratio; LMR, lymphocyte-to-monocyte ratio; ALRI, AST-to-lymphocyte ratio index.

Table S6. Univariable and multivariable results of binary logistic regression analysis for potential factors of early and late intrahepatic recurrence for patients who received RFA as initial treatment.

	**Early IHR**				**Late IHR**			
**Factors**	**Univariable**		**Multivariable**		**Univariable**		**Multivariable**	
	**OR (95 % CI)**	***p*-value**	**OR (95 % CI)**	***p*-value**	**OR (95 % CI)**	***p*-value**	**OR (95 % CI)**	***p*-value**
Sex (Male)	1.597 (1.226–2.080)	0.001	1.901 (1.402–2.579)	<0.001	1.435 (1.071–1.922)	0.016	1.610 (1.165–2.227)	0.004
Age (>60)	0.951 (0.755–1.197)	0.669			0.816 (0.630–1.056)	0.123		
Viral status (Viral)	0.824 (0.596–1.139)	0.241			1.361 (0.936–1.977)	0.106		
AST (>40)	1.831 (1.450–2.313)	<0.001			1.553 (1.198–2.013)	0.001		
ALT (>40)	1.723 (1.347–2.205)	<0.001			1.502 (1.142–1.977)	0.004		
ALBI grade (2–3)	2.060 (1.624–2.614)	<0.001	1.509 (1.128–2.020)	0.006	1.717 (1.318–2.236)	<0.001	1.446 (1.051–1.991)	0.024
Child-Pugh score (6–9)	1.617 (1.241–2.106)	<0.001			1.580 (1.180–2.116)	0.002		
AFP (>100)	1.612 (1.184–2.193)	0.002	1.565 (1.106–2.216)	0.011	1.252 (0.880–1.781)	0.211		
n of tumor (2–3)	2.198 (1.566–3.085)	<0.001	1.693 (1.102–2.600)	0.016	1.239 (0.827–1.858)	0.299		
Tumor size (>5cm)	1.885 (0.170–20.847)	0.605			-	-		
BCLC stage (A)	2.011 (1.590–2.542)	<0.001	1.431 (1.056–1.941)	0.021	1.529 (1.178–1.985)	0.001		
Platelet (≤150,000)	1.841 (1.390–2.439)	<0.001			1.446 (1.068–1.958)	0.017		
ANC (≤2,500)	1.673 (1.327–2.109)	<0.001			1.229 (0.953–1.585)	0.113	1.482 (1.077–2.038)	0.016
ALC (>1,200)	1.086 (0.838–1.406)	0.533			0.961 (0.724–1.276)	0.784		
AMC (>500)	1.074	0.604			1.114	0.477		

	(0.821–1.405)				(0.827–1.500)			
NLR (>1.0)	1.325 (0.992–1.770)	0.057			1.245 (0.901–1.720)	0.183		
PLR (≤65.0)	1.497 (1.191–1.882)	0.001	1.584 (1.196–2.097)	0.001	1.484 (1.150–1.915)	0.002	1.546 (1.142–2.095)	0.005
LMR (≤4.0)	1.076 (0.853–1.357)	0.539			1.046 (0.808–1.355)	0.731		
ALRI (<15.0)	2.069 (1.564–2.738)	<0.001	2.316 (1.639–3.271)	<0.001	1.590 (1.177–2.149)	0.003	2.010 (1.387–2.913)	<0.001
Change in NLR (>1.1 fold)	3.173 (2.493–4.039)	<0.001	2.114 (1.578–2.832)	<0.001	2.953 (2.264–3.852)	<0.001	2.247 (1.617–3.120)	<0.001
Change in PLR (>1 fold)	3.572 (2.810–4.540)	<0.001	2.983 (2.235–3.980)	<0.001	3.069 (2.358–3.995)	<0.001	2.336 (1.714–3.183)	<0.001
Change in LMR (≤1 fold)	3.052 (2.403–3.877)	<0.001	2.010 (1.516–2.665)	<0.001	2.790 (2.143–3.631)	<0.001	1.914 (1.405–2.606)	<0.001
Change in ALRI (>1.1 fold)	1.381 (1.097–1.737)	0.006			1.226 (0.949–1.583)	0.119		

RFA, radiofrequency ablation; AST, aspartate transaminase; ALT, alanine transferase; ALBI, albumin-bilirubin; AFP, alpha-fetoprotein; N, number; BCLC, Barcelona Clinic Liver Cancer; ANC, absolute neutrophil count; ALC, absolute lymphocyte count; AMC, absolute monocyte count; NLR, neutrophil-to-lymphocyte ratio; PLR, platelet-to-lymphocyte ratio; LMR, lymphocyte-to-monocyte ratio; ALRI, AST-to-lymphocyte ratio index.

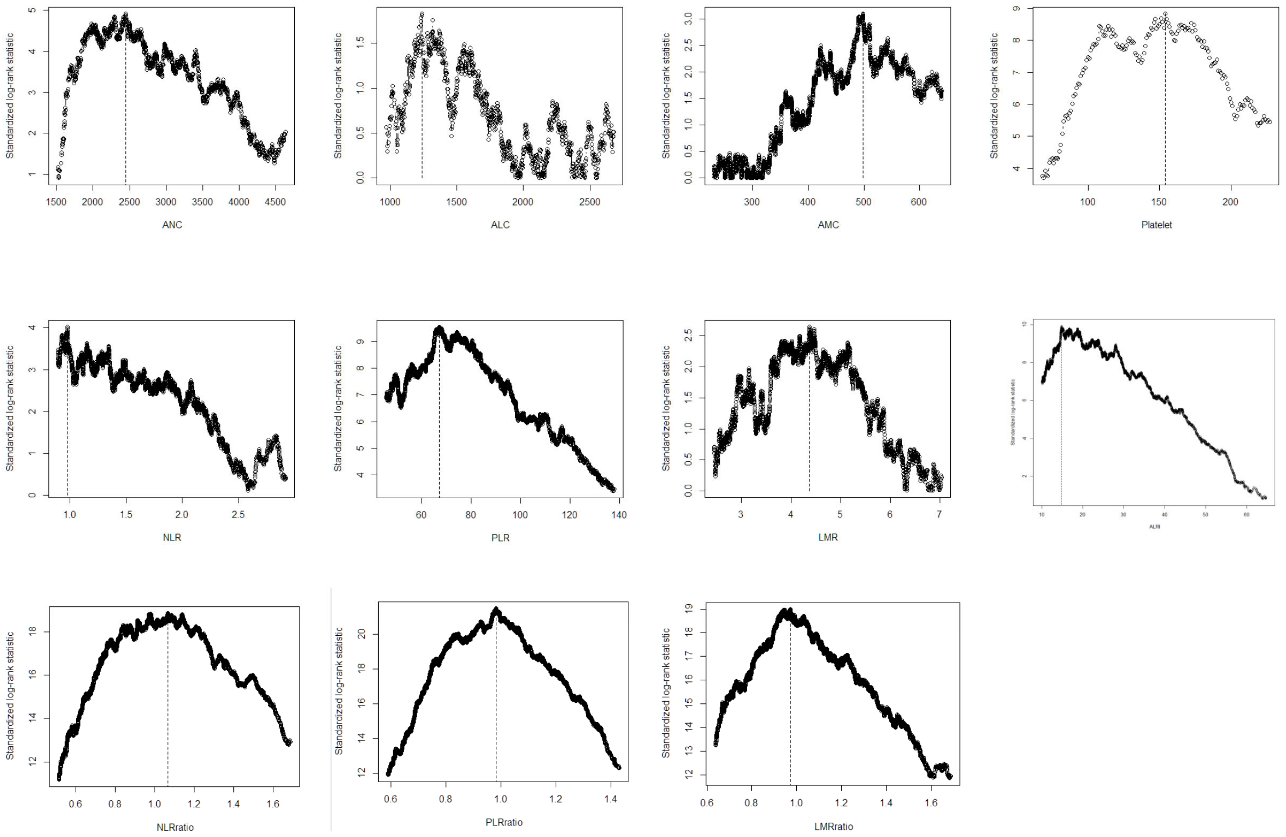

Figure S1. Results of maximally selected chi-square statistics to find optimal cut-off value of SIMs

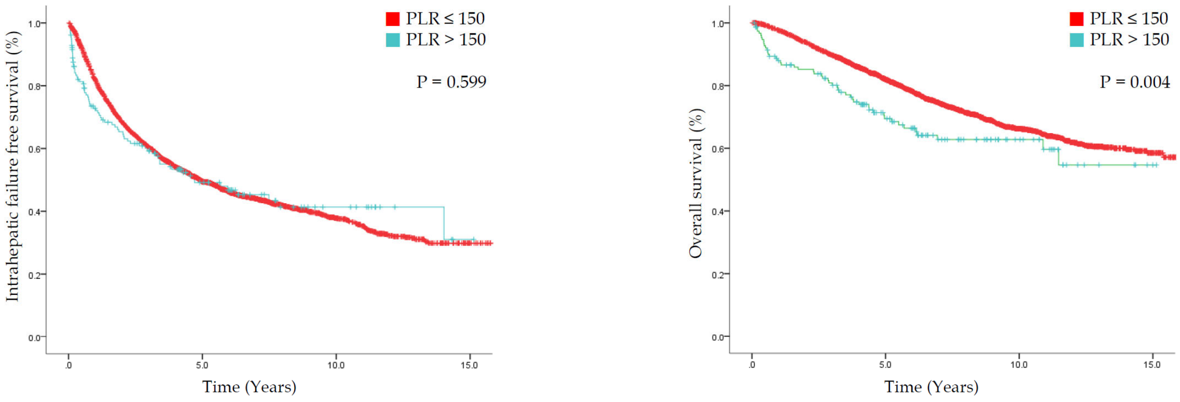

Figure S2. IHFFS and OS stratified by PLR 150, which is generally considered significant.
